# Characterization of mesenchymal stem cells in pre-B acute lymphoblastic leukemia

**DOI:** 10.3389/fcell.2023.1005494

**Published:** 2023-01-20

**Authors:** Anastasia M. Hughes, Vincent Kuek, Joyce Oommen, Grace-Alyssa Chua, Maria van Loenhout, Sebastien Malinge, Rishi S. Kotecha, Laurence C. Cheung

**Affiliations:** ^1^ Leukaemia Translational Research Laboratory, Telethon Kids Cancer Centre, Telethon Kids Institute, Perth, WA, Australia; ^2^ Curtin Medical School, Curtin University, Perth, WA, Australia; ^3^ School of Medicine, University of Western Australia, Perth, WA, Australia; ^4^ Department of Clinical Haematology, Oncology, Blood and Marrow Transplantation, Perth Children’s Hospital, Perth, WA, Australia; ^5^ Curtin Health Innovation Research Institute, Curtin University, Perth, WA, Australia

**Keywords:** pre-B cell acute lymphoblastic leukemia, leukemia, mesenchymal stem cell, bone marrow microenvironment, bone marrow

## Abstract

Components of the bone marrow microenvironment (BMM) have been shown to mediate the way in which leukemia develops, progresses and responds to treatment. Increasing evidence shows that leukemic cells hijack the BMM, altering its functioning and establishing leukemia-supportive interactions with stromal and immune cells. While previous work has highlighted functional defects in the mesenchymal stem cell (MSC) population from the BMM of acute leukemias, thorough characterization and molecular profiling of MSCs in pre-B cell acute lymphoblastic leukemia (B-ALL), the most common cancer in children, has not been conducted. Here, we investigated the cellular and transcriptome profiles of MSCs isolated from the BMM of an immunocompetent BCR-ABL1^+^ model of B-ALL. Leukemia-associated MSCs exhibited reduced self-renewal capacity *in vitro* and significant changes in numerous molecular signatures, including upregulation of inflammatory signaling pathways. Additionally, we found downregulation of genes involved in extracellular matrix organization and osteoblastogenesis in leukemia-associated MSCs. This study provides cellular and molecular insights into the role of MSCs during B-ALL progression.

## 1 Introduction

Precursor-B cell acute lymphoblastic leukemia (B-ALL) is the most common cancer in children. While an improved understanding of the pathophysiology of this disease has led to increasingly effective and optimized treatment strategies, certain high-risk B-ALL subtypes remain difficult to cure (Inaba and Mullighan, 2020). Relapsed or refractory disease, as well as the presence of high-risk genetic mutations in blast cells, such as the *BCR-ABL1* translocation or *KMT2A*-rearangements, are risk factors for poor outcomes (Inaba and Mullighan, 2020). As a result, extensive pre-clinical research is urgently required to identify new treatment strategies.

Hematopoiesis, the process through which lymphoid and myeloid blood cells are formed, is intimately regulated by stromal and immune cell populations that reside within the bone marrow (BM). These cellular constituents, along with blood vessels and extracellular matrix (ECM) proteins, form the bone marrow microenvironment (BMM). The spatial organization of cells and vasculature in this region create distinct ‘niches’ that carry out different roles during hematopoiesis (Pinho and Frenette, 2019). The importance of these BM niches in regulating not only healthy hematopoiesis, but also hematological malignancies, has been extensively reviewed (Mendez-Ferrer et al., 2020; Hughes et al., 2022). Leukemic cells can exploit and remodel the BMM to favor leukemia growth, survival and treatment resistance *via* various mechanisms (Mendez-Ferrer et al., 2020; Dander et al., 2021; Hughes et al., 2022). Thus, an in-depth understanding of the interactions between leukemic cells and the BMM may uncover novel therapeutic targets for treatment.

An essential component of the BMM is the mesenchymal stem cell (MSC). MSCs can give rise to stromal cells including adipocytes, osteoblasts and chondrocytes, and provide regulatory cues for hematopoiesis *via* adhesion molecules and soluble factors (Wei and Frenette, 2018; Kandarakov et al., 2022). However, numerous alterations have been described in mesenchymal stem/stromal cells following leukemic disease in the BM. In myeloid malignancies, MSCs have been found to exhibit molecular and cellular level alterations that establish a microenvironment favoring leukemogenesis at the expense of healthy hematopoiesis (Geyh et al., 2016; Battula et al., 2017; von der Heide et al., 2017; Baryawno et al., 2019; Borella et al., 2021; Zhang et al., 2021). Additionally, MSCs have been shown to establish a chemoprotective sanctuary for acute myeloid leukemia (AML) cells, providing metabolic and antioxidant support (Forte et al., 2020). Similarly, in the T-cell ALL (T-ALL) BMM, MSCs exhibit impaired differentiation potential, increased cellular senescence and reduced hematopoietic progenitor supportive function (Lim et al., 2016).

While less is known about the BMM of B-ALL, studies have shown that MSCs promote engraftment, survival and chemoresistance of B-ALL cells (Iwamoto et al., 2007; Nwabo Kamdje et al., 2011; Frolova et al., 2012; Jacamo et al., 2014; Hu et al., 2015; Mallampati et al., 2015; Polak et al., 2015; Burt et al., 2019; Portale et al., 2019; Yu et al., 2019; Ruiz-Aparicio et al., 2020; Tarighat et al., 2021). Furthermore, B-ALL cells are capable of disrupting the proliferation, differentiation, protein expression, signaling pathway activation and hematopoietic-supporting capabilities of MSCs (Conforti et al., 2013; Jacamo et al., 2014; van den Berk et al., 2014; Vicente Lopez et al., 2014; Polak et al., 2015; Balandran et al., 2016; de Rooij et al., 2017; Vernot et al., 2017; Ma et al., 2019; Portale et al., 2019; Yu et al., 2019; Verma et al., 2020; Vanegas et al., 2021). Although, these studies provide a clear indication that MSCs are important components of the B-ALL BMM, the molecular-level alterations in B-ALL-associated MSCs have not been comprehensively assessed. Furthermore, little is known about the role of MSCs in the BMM of the high-risk BCR-ABL1^+^ B-ALL subtype.

Previous characterization of B-ALL-associated MSCs has largely relied on *in vitro* co-culture assays or *in vivo* experiments using immunocompromised, patient-derived xenograft mouse models of B-ALL. Here, we used our previously characterized immune competent, syngeneic mouse model of BCR-ABL1^+^ B-ALL to further explore the cellular and molecular alterations of BM MSC populations (Cheung et al., 2018; Anderson et al., 2020).

## 2 Methods

### 2.1 Murine model of BCR-ABL1^+^ B-ALL

Female C57BL/6J mice aged between 7 to 9 weeks were intravenously injected with 1000 B-ALL cells (PER-M60 cells) carrying MSCV-BCR-ABL1-IRES-mCherry retrovirus *via* tail vein (Cheung et al., 2018). Non-leukemic mice injected with 200 μl of plain phosphate-buffered saline (PBS) were used as controls. Mice were euthanized at onset of disease symptoms. For euthanasia, mice were first anesthetized by isoflurane inhalation (3%), followed by cervical dislocation. BM disease burden in mice ranged between 40%–80% leukemic blasts, assessed by the percentage of mCherry^+^ cells by flow cytometry. All experiments were approved by the Animal Ethics Committee, Telethon Kids Institute (AEC#311 and #330).

### 2.2 Isolation and staining of primary MSCs from long bones

Primary MSCs were isolated from the long bones of control or B-ALL mice according to a previously published protocol with modifications (Houlihan et al., 2012). Following mice euthanasia, tibias and femurs were excised, and BM was flushed out of the medullary cavity and discarded. The long bones were cut into small fragments with scissors, washed thrice with PBS, and incubated with 1.5 mg/ml Collagenase Type 4 (Worthington Biochemical Corp.) and 0.1 mg/ml DNase I (Sigma-Aldrich) in PBS supplemented with 10% fetal calf serum (FCS) at 37°C for 60 min under agitation. Bone digests were filtered through sterile 100 μm cell strainers, and bone fragments were crushed gently using a mortar and pestle in PBS with 5% FCS to detach MSCs. Cells were washed from bone fragments with PBS, strained, and pooled into the bone digests. This was repeated 6 times to maximize the cell yield. Cells were pelleted and treated with Red Blood Cell Lysis Buffer (BD Biosciences), followed by washing and resuspension in 5% FCS/PBS. Cell suspensions were either stained with appropriate antibodies for flow cytometry analysis or fluorescence-activated cell sorting (FACS), or first cultured *in vitro* to increase the number of cells.

For staining of MSCs, cells were first stained with BD Horizon Fixable Viability Stain 700 to exclude non-viable cells, followed by CD45-PerCP-Cy5.5 and Ter119-PerCP-Cy5.5 to exclude hematopoietic cells and CD31-FITC to exclude endothelial cells. PαS MSCs were identified using Sca-1-BV510 and PDGFRα-APC (Morikawa et al., 2009). The gating strategy is shown in Supplementary Figure S1A. All antibodies other than PDGFRα-APC (eBioscience) were purchased from BD Biosciences. Flow cytometry was performed using a BD Fortessa and FACS was performed using a BD FACSAria. Flow cytometry data was analyzed using FlowJo V10.5.3 software (BD Biosciences).

### 2.3 Culture of primary MSCs

For culture and maintenance of primary MSCs, cells derived from murine long bones were incubated at 37°C and 5% CO_2_ (unless stated otherwise) for 7–10 days in MesenCult media (Stem Cell Technologies) supplemented with 100 Units/ml penicillin, 100 μg/ml streptomycin (Thermo Fisher Scientific), 1% L-glutamine and 0.1% MesenPure (Stem Cell Technologies). Media change was performed every 2–3 days. Upon reaching confluence, cells were detached enzymatically using TrypLE Express Enzyme (Thermo Fisher Scientific), pelleted, stained and isolated by FACS for use in functional assays. Only freshly isolated primary MSCs, or primary MSCs at passage 1 (P1) were used for functional assays.

### 2.4 Colony-forming unit-fibroblast (CFU-F) assay

Freshly isolated MSCs were seeded at a density of 800 cells per well in a 6-well plate and cultured for 14 days in alpha-MEM medium supplemented with 10% FCS, 100 Units/ml penicillin, 100 μg/ml streptomycin, 1% L-glutamine, 55 μM 2-mercaptoethanol, 1% non-essential amino acid mix and 1% 100 mM sodium pyruvate solution (MP Biomedicals). After 14 days, media was removed, and cells were processed and stained with Giemsa (Sigma-Aldrich) as previously described (Lim et al., 2016). A colony was defined as 50 or more cells. The number of colonies were counted in each well using an Olympus IX71 microscope.

### 2.5 Bromodeoxyuridine (BrdU) proliferation assay

The *in vivo* proliferation rate of MSCs was assessed by a BrdU incorporation assay. BrdU was given to control and B-ALL mice on day 3 post B-ALL cell injection according to a previously published protocol (Zhou et al., 2014). Mice were sacrificed 18 days post leukemia cell injection. MSCs were stained for surface markers, followed by staining of BrdU using the FITC-BrdU kit (BD Biosciences) according to manufacturer’s instructions. Samples were analyzed by flow cytometry. The gating strategy is shown in Supplementary Figure S1B. Differentiation assay methodology can be found in the Supplementary Material.

### 2.6 MSC and B-ALL cell co-culture

To detect B-ALL cells by bioluminescence, we transduced the PER-M60 B-ALL cell line with a firefly luciferase reporter construct (MSCV-ires-pacLUC2) and purified luciferase-expressing PER-M60 cells by puromycin selection (Endersby et al., 2018). MSCs were seeded at 15000 cells per well into a 96-well plate in alpha-MEM media and allowed to adhere overnight. The following day, 3000 luciferase-expressing B-ALL cells were seeded in co-culture with MSCs. Following co-culture for 3 days, D-luciferin (PerkinElmer) was added to each well at a final concentration of 150 μg/ml and luciferase bioluminescent signal in each well was measured on a CLARIOstar plate reader (BMG Labtech). Leukemic cell numbers from each well were calculated from a bioluminescence standard curve and analyzed accordingly.

### 2.7 MSC and LSK cell co-culture

MSCs were seeded into a 96-well plate at 17500 cells per well in alpha-MEM medium and allowed to adhere overnight. Lineage^−^, Sca-1^+^, c-Kit^+^ (LSK) hematopoietic progenitor cells were sorted the following day. To isolate LSK cells, five 8-week-old, healthy mice were sacrificed and femurs, tibias, ilia and humeri were excised. BM was flushed out of the medullary cavity with 2% FCS/PBS. BM cells were pelleted and treated with Red Blood Cell Lysis Buffer. Cells were washed with 2% FCS/PBS, followed by enrichment of hematopoietic progenitor cells using the EasySep Mouse Hematopoietic Progenitor Isolation Kit (Stem Cell Technologies) according to manufacturer’s instructions. Cells were then counted using a hemocytometer. Dead cells were excluded using BD Horizon Fixable Viability Stain 700 and cells were stained with CD117-PerCP-Cy5.5, Streptavidin-APC-Cy7 (for removal of any remaining cells bound by the biotinylated antibody cocktail) and Sca-1-PE-Cy7. LSK cells were isolated by FACS. The gating strategy is shown in Supplementary Figure S1C. All antibodies were purchased from BD Biosciences.

LSK cells were seeded in wells with MSCs at 9000/well in alpha-MEM medium. After a 3 days co-culture, suspension cells were removed from the wells. Adherent cells were detached enzymatically using TrypLE Express Enzyme and pooled with suspension cells. Cells were pelleted and stained with CD45-FITC. CD45^+^ hematopoietic cells were enumerated and isolated by FACS. The multilineage potential of these hematopoietic cells was then assessed using a colony-forming assay. Briefly, 5000 CD45^+^ cells were seeded in 35 mm dishes in complete MethoCult medium (M3434, Stem Cell Technologies). After 7 days, the number of colony-forming unit-granulocyte, erythroid, macrophage, megakaryocyte (CFU-GEMM), colony-forming unit-granulocyte, macrophage (CFU-GM), colony-forming unit-macrophage (CFU-M), colony-forming unit-granulocyte (CFU-G), colony-forming unit-megakaryocyte (CFU-Mk) and burst-forming unit-erythroid (BFU-E) were counted using an Olympus IX71 microscope. Colonies were identified and analyzed according to manufacturer’s instructions.

### 2.8 RNA sequencing of MSCs

MSCs were extracted and isolated from the long bones of control or leukemia mice (pooled from 4 to 7 mice per sample) *via* FACS as described above, yielding between 12000 to 60000 MSCs per sample. Two biological replicates were collected for both control and leukemia-associated MSCs (L-MSCs). Following sorting, total RNA from MSCs was extracted using the RNeasy Micro Kit (Qiagen). Samples were then sent to BGI, Hong Kong for sequencing. RNA concentration, quality and integrity were assessed using an Agilent 2100 BioAnalyzer to ensure a RNA integrity number of 6.5 or above. RNA amplification was performed using the SMART-Seq v4 Ultra Low Input RNA Kit (Takara Bio). Quality control of amplification products was conducted prior to tagmentation-based library construction. Circularization and library quality control was performed prior to 100 bp paired-end RNA sequencing using the BGISEQ-500 platform. Sequencing data is available *via* the Gene Expression Omnibus (GEO) database under the accession number GSE208719.

Data processing and analysis was performed by BGI, Hong Kong. Quality control and filtering of data was conducted to remove reads containing the adaptor, unknown base N content greater than 5% and low-quality reads using SOAPnuke software (v1.5.2, BGI) (Chen et al., 2018). Clean reads ranged between 57 and 74M per sample. Clean reads were aligned to the reference genome and genes (Mus_musculus, NCBI, version: GCF_000001635.26_GRCm38. p6) using HISAT2 (v2.0.4) and Bowtie2 (v2.2.5) respectively (Langmead and Salzberg, 2012; Kim et al., 2015). The gene expression level for each sample was calculated using RSEM (v1.2.8) (Li and Dewey, 2011). Principle component analysis was conducted using BGI’s Dr Tom analysis software on FPKM values standardized by z-score. Identification of differentially expressed genes (DEGs) (|Log2FC|≥1, q-value≤0.05) between control and L-MSCs was performed using the DEseq2 method (Love et al., 2014). Gene set enrichment analysis (GSEA) using the Kyoto Encyclopedia of Genes and Genomes (KEGG) database and gene ontology (GO) enrichment analysis was performed using Dr. Tom. For GSEA, a |normalized enrichment score (NES)|≥1, nominal (NOM) *p*-value ≤0.05 and false discovery rate (FDR) q-value ≤0.25 were used as threshold values.

### 2.9 Quantitative reverse transcription polymerase chain reaction (PCR)

RNA was extracted from MSCs as described above, followed by cDNA synthesis using SuperScript VILO Master Mix (Thermo Fisher Scientific). Quantitative PCR (qPCR) was performed on a QuantStudio 7 Flex Real-Time PCR System (Applied Biosystems) using the TaqMan Fast Advanced Master Mix (Thermo Fisher Scientific) and the following TaqMan gene expression assays: mouse *Thbs1* (Mm00449032_g1), mouse *Dpep1* (Mm00514592_m1), mouse *C3* (Mm01232779_m1), mouse *Cfh* (Mm01299248_m1), mouse *Col1a1* (Mm00801666_g1), mouse *Bmp6* (Mm01332882_m1) and mouse *Gapdh* (Mm99999915_g1) (Thermo Fisher Scientific). Relative expression levels were calculated using the ΔΔCT method, normalized to *Gapdh* for each individual sample (Livak and Schmittgen, 2001).

### 2.10 Statistical analysis

Statistical analyses were carried out using GraphPad Prism version 8.1.1. Two-tailed unpaired Student’s t-test was performed for comparison between groups. Results are presented as mean ± SEM and a *p*-value ≤0.05 was deemed statistically significant.

## 3 Results

### 3.1 Leukemia-associated MSCs exhibit reduced self-renewal potential

**FIGURE 1:**
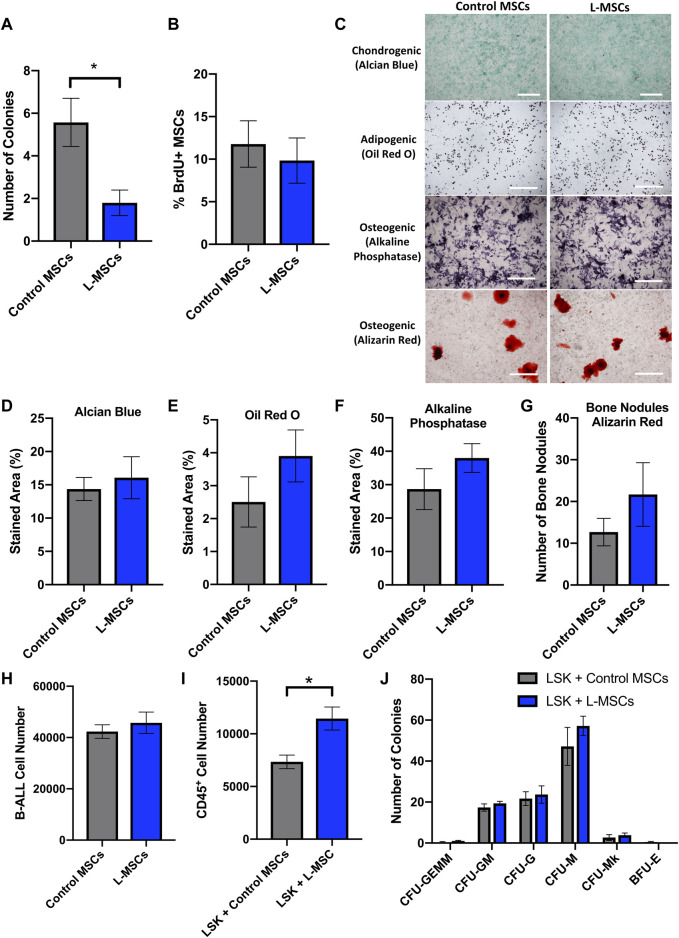
Cellular characterization of L-MSCs reveals reduced self-renewal capacity and increased potential to support the expansion of LSK cells *in vitro*. **(A)** Quantification of the number of MSC CFU-F colonies (>50 cells) per well after 14 days in culture. Data were collated from three independent experiments (n = 7). **(B)** Quantification of BrdU^+^ MSCs isolated from the long bones of control or B-ALL mice. Data were collated from two independent experiments (n = 7). **(C)** Representative images showing Alcian blue, Oil Red O, alkaline phosphatase and Alizarin Red-stained cells following culture of control or L-MSCs in chondrogenic, adipogenic and osteogenic differentiation media. **(D)** Quantification of Alcian blue-stained area following chondrogenic differentiation of MSCs for 20-22 days. **(E)** Quantification of Oil Red O-stained area following adipogenic differentiation of MSCs for 6 days. **(F)** Quantification of alkaline phosphatase-stained area and **(G)** number of Alizarin Red-stained bone nodules following osteogenic differentiation of MSCs for 13 days and 26–28 days respectively. Data from differentiation assays are presented as mean ± SEM (n = 3 per group) and are representative of two independent experiments. **(H)** Number of luciferase-expressing B-ALL cells per well following a 3 days co-culture with control or L-MSCs (n = 3). **(I)** Number of hematopoietic (CD45^+^) cells per well after a 3 days co-culture with control or L-MSCs (control MSCs n = 4, L-MSCs n = 5). **(J)** Quantification of the number of CFU colonies formed by LSK cells post co-culture with control or L-MSCs (n = 3). This experiment has been repeated twice. Data presented are from a single experiment but is representative of both independent experiments. All images are at × 4 magnification and scale bars represent 500 μm. Error bars represent mean ± SEM. *p ≤ 0.05.

First, we utilized a CFU-F assay to assess whether the self-renewal potential of MSCs from leukemia-bearing mice was altered. L-MSCs, harvested from mice with BM leukemia burden exceeding 40%, formed significantly fewer colonies than control MSCs, demonstrating reduced self-renewal potential in the B-ALL BMM (Figure 1A). We further examined the *in vivo* proliferative potential of L-MSCs, as indicated by BrdU^+^ MSCs in a BrdU incorporation assay, with no significant difference identified compared to control MSCs (Figure 1B).

### 3.2 The differentiation potential of MSCs is not altered by B-ALL

Next, we performed a tri-lineage differentiation assay to assess the ability of L-MSCs to differentiate into chondrocytes, adipocytes and osteoblasts. MSCs from both healthy and leukemia-bearing mice successfully differentiated into chondrocytes, adipocytes and osteoblasts (Figure 1C), with no significant difference in differentiation capacity detected between the two groups (Figures 1D–F). Furthermore, osteoblasts derived from L-MSCs did not exhibit a significant difference in bone nodule formation when compared to the osteoblasts derived from control MSCs (Figure 1G). Thus, we conclude that the ability of L-MSCs to differentiate into stromal components of the BMM is not altered in this *in vitro* setting.

### 3.3 L-MSCs possess similar leukemogenic supportive capabilities *in vitro* compared to healthy MSCs

We next used an *in vitro* co-culture assay to investigate whether L-MSCs affect the proliferation of luciferase-expressing B-ALL cells. Here, we compared the proliferation of B-ALL cells cultured in the presence of MSCs that were isolated from the BMM of healthy or B-ALL-bearing mice. We found that the number of viable B-ALL cells after 3 days in co-culture remained similar between control and L-MSCs, indicating that they possess similar capabilities to support B-ALL cell growth *in vitro* (Figure 1H).

### 3.4 The hematopoietic supportive role of MSCs is altered by B-ALL

MSCs are important regulators of normal hematopoiesis and this function can be altered by leukemia (Conforti et al., 2013; Vicente Lopez et al., 2014; Lim et al., 2016; Pinho and Frenette, 2019). To assess the hematopoietic supportive ability of L-MSCs, we compared the growth of LSK cells containing hematopoietic stem and progenitor cells with MSCs derived from either healthy control or B-ALL mice. The growth of LSK cells over 3 days was significantly increased in the presence of L-MSCs compared to control MSCs (Figure 1I). Next, we examined the myelopoietic supportive role of L-MSCs *in vitro*. Colony-forming assays were performed to assess the differentiation potential of LSK cells into CFU-GEMM, CFU-GM, CFU-M, CFU-G, CFU-Mk and BFU-E after they were co-cultured with MSCs. The ability of L-MSCs in inducing myeloid commitment and differentiation did not differ significantly when compared to control MSCs (Figure 1J).

### 3.5 Molecular characterization of L-MSCs reveals alterations to inflammatory, ECM and osteogenic related processes

**FIGURE 2:**
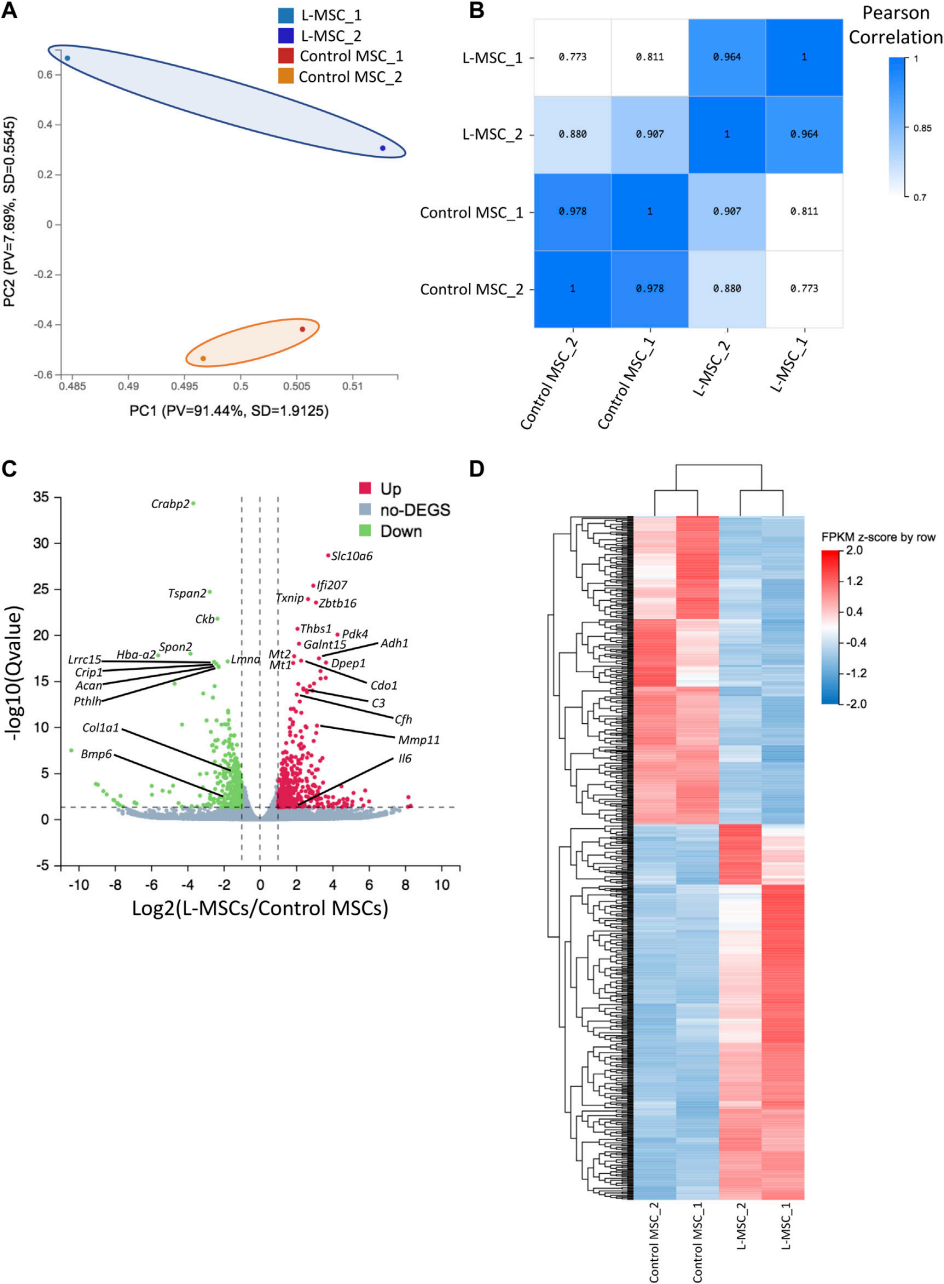
RNA sequencing reveals molecular changes in L-MSCs. **(A)** Principal component analysis based on row z-score of FPKM values for control and L-MSCs shows that the control and L-MSC group variable was separated by PC2 (n = 2, four to seven mice pooled per sample). **(B)** Sample correlation heatmap based on Pearson correlation coefficient identified that intergroup variability was greater than intragroup variability. **(C)** Volcano plot demonstrating the presence of 770 differentially expressed genes (DEGs) in L-MSCs compared to control MSCs (|Log2FC|≥1, q-value≤0.05). Green represents downregulated genes; red represents upregulated genes. **(D)** Unsupervised clustering of DEGs shown in the volcano plot. Heatmap is of FPKM values standardized by row z-score.

In addition to characterizing L-MSCs’ cellular functions and their impact on leukemia and hematopoietic cells, we elucidated changes occurring at the transcriptomic level *via* RNA-sequencing of MSCs harvested from control or leukemia mice. Principle component analysis highlighted that control and L-MSCs are transcriptionally distinct, with B-ALL and control MSCs separated by principal component 2 (Figure 2A) and a sample correlation analysis confirming that intergroup variability was greater than intra-group variability (Figure 2B). Differential expression analysis identified a total of 770 DEGs, of which 423 genes were upregulated and 347 genes were downregulated in L-MSCs when compared to control MSCs (Figures 2C, D; Supplementary Tables S1, S2).

**FIGURE 3:**
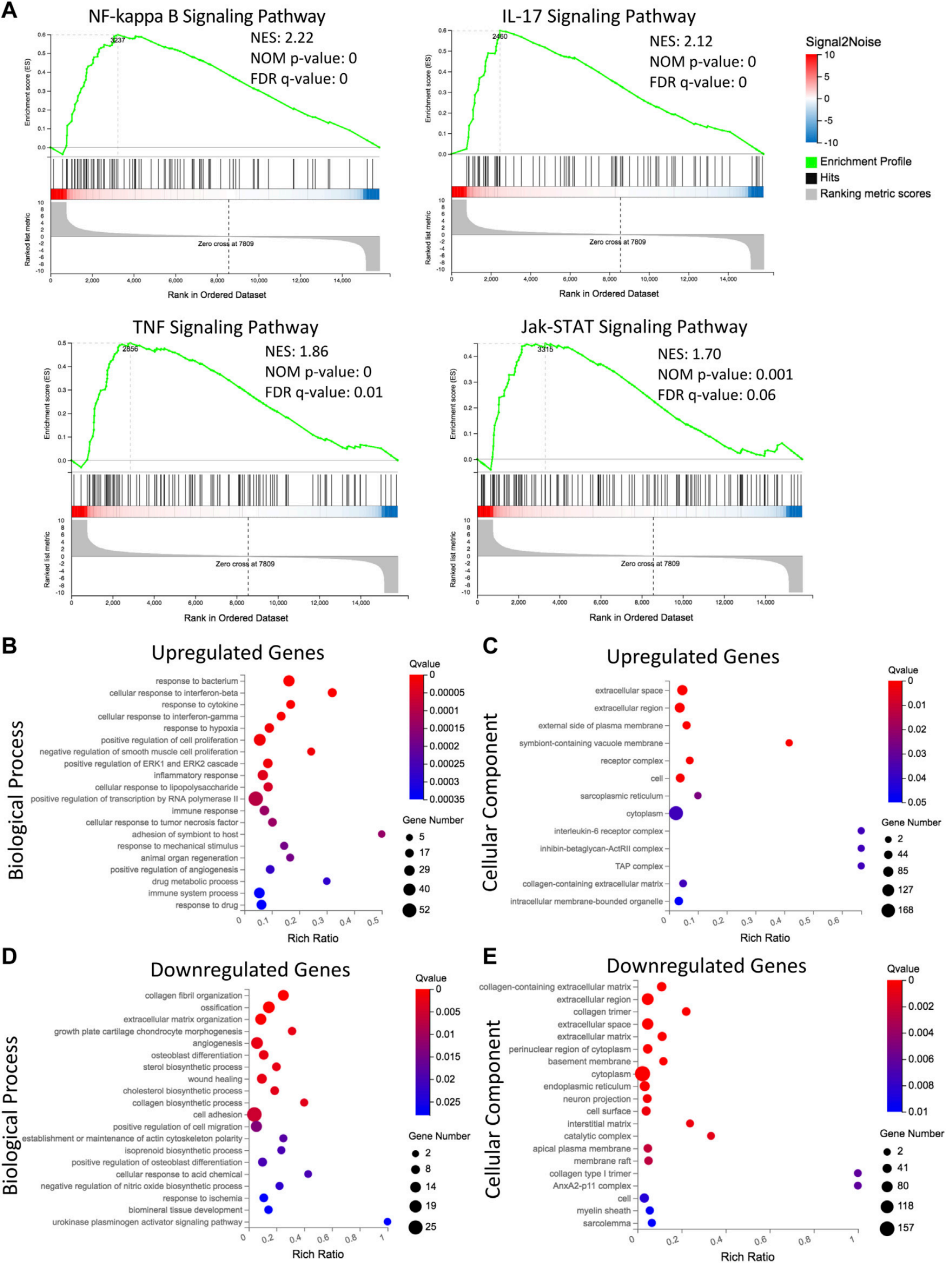
Transcriptomic analysis reveals distinct upregulation of immune and inflammatory related signaling, and downregulation of extracellular matrix and osteoblastogenesis related processes in L-MSCs. **(A)** Gene set enrichment analysis showing gene sets that are upregulated in L-MSCs (NF-κB, IL-17, TNF and Jak-STAT signaling pathways). Normalized enrichment score (NES), nominal (NOM) *p*-value and false discovery rate (FDR) q-value are shown for each plot. **(B)** Gene ontology (GO) analysis of biological processes enriched in the 423 genes upregulated by L-MSCs. The top 20 significantly enriched biological processes are displayed (based on q-value≤0.05). **(C)** GO analysis of cellular components enriched in the 423 upregulated genes in L-MSCs. The top 13 significantly enriched cellular components are displayed. **(D)** GO analysis of biological processes enriched in the 347 genes downregulated in L-MSCs. The top 20 significantly enriched biological processes are displayed. **(E)** GO analysis of cellular components enriched in the 347 downregulated genes in L-MSCs. The top 20 significantly enriched cellular components are displayed.

**FIGURE 4:**
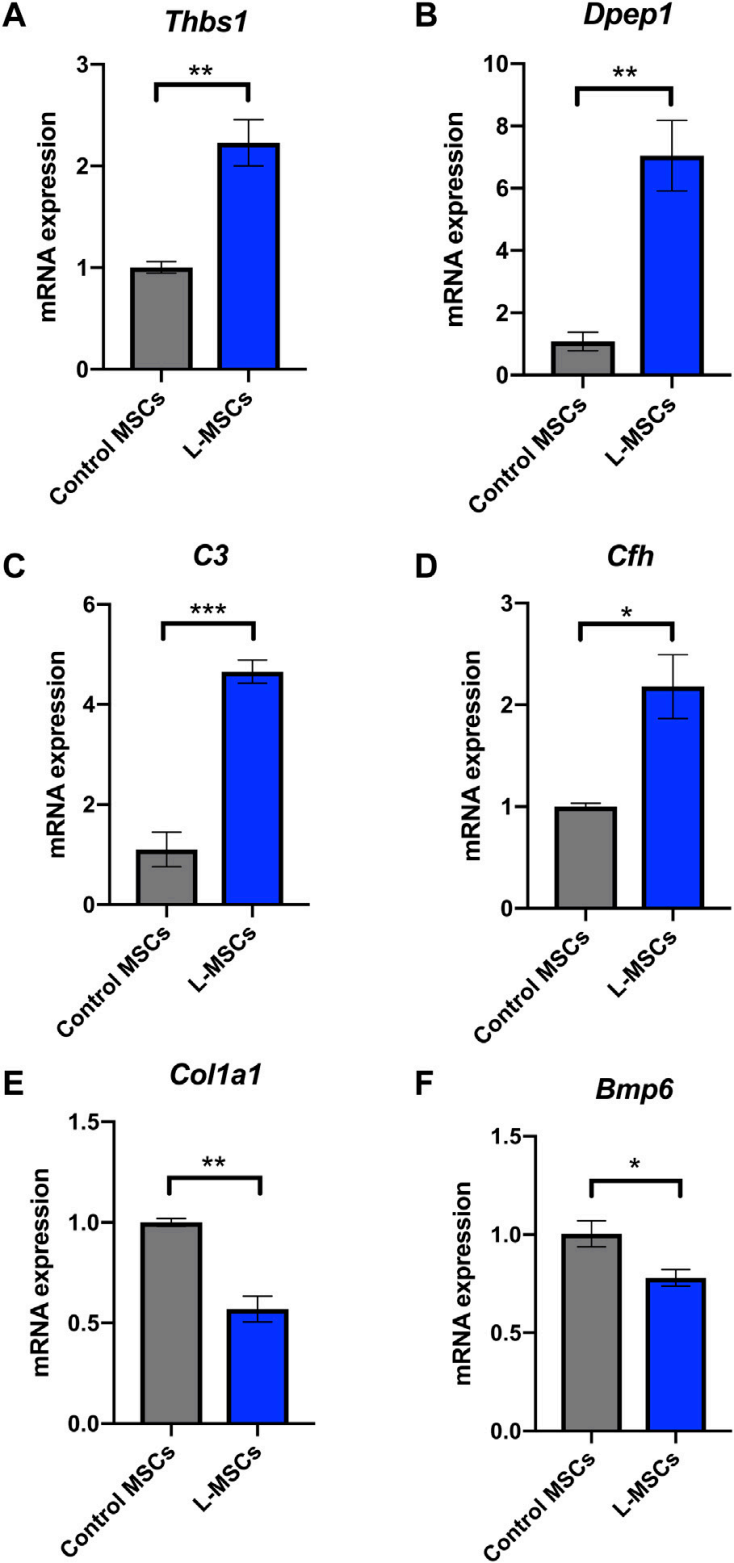
Inflammatory response related genes *Thbs1*, *Dpep1*, *C3* and *Cfh* are upregulated in L-MSCs while osteoblast differentiation related genes *Col1a1* and *Bmp6* are downregulated in L-MSCs. Mean expression of inflammatory response related genes **(A)** *Thbs1*, **(B)** *Dpep1*, **(C)** *C3* and **(D)** *Cfh* in L-MSCs relative to that in control MSCs. Mean expression of osteoblast differentiation related genes **(E)** *Col1a1* and **(F)** *Bmp6* in L-MSCs relative to that in control MSCs. Control MSCs n = 3, L-MSCs n = 4. Data are presented as mean ± SEM. *p ≤ 0.05, **p ≤ 0.01, ***p ≤ 0.001.

To assess the biological relevance of DEGs, we conducted GSEA using the KEGG pathway database. We identified an enrichment of inflammatory related pathways such as NF-kappa B (NF-κB), interleukin-17 (IL-17), tumor necrosis factor (TNF) and Jak-STAT in L-MSCs (Figure 3A). Furthermore, GO enrichment analysis revealed biological processes enriched in upregulated gene sets included ‘cellular response to interferon-beta’, ‘cellular response to interferon-gamma’, ‘inflammatory response’, ‘immune response’, ‘cellular response to TNF’ and ‘immune system processes’ (Figure 3B). GO analysis further revealed that the most enriched cellular components were the ‘extracellular region’, ‘extracellular space’ and ‘external side of plasma membrane’ (Figure 3C). To validate the upregulation of inflammatory gene signatures in L-MSCs we examined the expression of the four most significantly upregulated genes in the ‘inflammatory response’ process identified by our GO analysis. By qPCR we identified that the expression of thrombospondin 1 (*Thbs1*), dipeptidase 1 (*Dpep1*), complement C3 (*C3*) and complement factor H (*Cfh*) were significantly increased in L-MSCs compared to control MSCs (Figures 4A–D).

GO enrichment analysis of genes downregulated in L-MSCs identified biological processes involved in ‘ECM organization’, ‘collagen fibril organization’ and ‘collagen biosynthetic process’ (Figure 3D). In line with this, GO analysis of cellular components identified that downregulated gene sets were enriched in the ‘collagen-containing ECM’, ‘extracellular region’ and ‘extracellular space’ (Figure 3E). Finally, downregulated genes were also enriched for osteogenic-related processes including ‘ossification’, ‘osteoblast differentiation’, ‘positive regulation of osteoblast differentiation’ and ‘angiogenesis’ which intimately couples osteogenesis (Figure 3D). These results indicate that major biological processes downregulated at the molecular level in L-MSCs appear to affect ECM regulation and osteoblast differentiation. To validate the downregulation of osteogenic related genes in L-MSCs we assessed the expression of two of the most significantly downregulated genes in the ‘osteoblast differentiation’ process identified by our GO analysis. By qPCR we showed that both collagen type 1 alpha 1 (*Col1a1*) and bone morphogenetic protein 6 (*Bmp6*) were expressed at significantly lower levels in L-MSCs than control MSCs (Figures 4E,F).

## 4 Discussion

Current treatment options for leukemia largely rely on therapies that directly target malignant cells. In recent years, the BMM of leukemia has been highlighted as a critical therapeutic target due to the propensity of leukemic cells to hijack and remodel normal BM niches into a “sanctuary” that facilitates chemoresistance and immune escape. Therefore, normalizing BMM function or blocking leukemia-BMM interactions may create a less hospitable environment for leukemic cells and could thus lead to improved therapies for the treatment of high-risk leukemia.

Our *in vitro* data indicates that most cellular functions of L-MSCs do not differ significantly when compared to control. Interestingly, L-MSCs showed a reduction in self-renewal capacity, as demonstrated by the formation of fewer CFU-Fs *in vitro.* This has previously been observed in B-ALL and T-ALL, with our data providing further evidence that ALL cells can inhibit the self-renewal capacity of MSCs (Balandran et al., 2016; Lim et al., 2016). While our study did not detect any significant changes in the percentage of proliferating MSCs through BrdU incorporation *in vivo*, other studies have observed decreased proliferation of MSCs and stromal cells in B-ALL (Conforti et al., 2013; Vicente Lopez et al., 2014; Balandran et al., 2016; Zanetti et al., 2020). A reduced proliferative potential has been attributed to the induction of cellular senescence in MSCs in the ALL BMM (Lim et al., 2016; Bonilla et al., 2019; Vanegas et al., 2021). These studies were conducted in different subtypes of ALL, suggesting that the MSCs in different subtypes of ALL may have different proliferation profiles. We also examined the effect of MSCs on leukemia cell number and viability following a 3 days co-culture and observed that both control MSCs and L-MSCs have a similar effect on leukemia cell growth *in vitro*.

Our previous study demonstrated that the number of osteoblasts and trabecular bone mass are significantly reduced in leukemia-bearing mice (Cheung et al., 2018). However, it is unclear whether this is caused by impaired osteogenic potential of MSCs during leukemogenesis. In this study, we have shown that L-MSCs retained their tri-lineage differentiation potential *in vitro* and we did not observe significant changes in osteogenic potential between normal MSCs and L-MSCs. Our results appear to be consistent with findings in other B-ALL subtypes (Balandran et al., 2016; Zanetti et al., 2020). In contrast, one study demonstrated a modest reduction in osteogenic differentiation potential of B-ALL-associated MSCs (Vanegas et al., 2021). It is possible that the discrepancy in findings is due to variability between cell lines and primary samples used. While it appears that the osteogenic potential of MSCs is not affected by leukemia development in an *in vitro* culture setting, the transcriptomic analysis of DEGs indicated an osteo-lineage defect in L-MSCs. Specifically, GO analysis of biological processes identified a downregulation in genes associated with osteoblast differentiation and osteogenesis in L-MSCs which we validated by qPCR. It is plausible that the impact on osteoblast differentiation is highly dependent on complex crosstalk with other cellular components of the BM niche. For instance, the B-ALL niche is known to be rich in pro-inflammatory cytokines and the pro-inflammatory cytokines TNF and IL-17 are known to inhibit osteoblast differentiation in MSCs *via* activation of the NF-κB signaling pathway (de Vasconcellos et al., 2011; Chang et al., 2013; Balandran et al., 2016). Our GSEA data confirmed that genes in these pathways are upregulated in L-MSCs, thus potentially contributing to a reduction in osteoblast formation. NF-κB activation can also promote the expression of interleukin-6 (IL-6), which is a pro-inflammatory cytokine known to promote osteoclastogenesis and inhibit osteoblastogenesis and is therefore commonly associated with various bone diseases such as rheumatoid arthritis and osteoporosis (Libermann and Baltimore, 1990; Harmer et al., 2018). Importantly, the expression of *Il6* was significantly increased in L-MSCs in our study (Log2FC = 2.12), thus could be contributing to the bone loss observed in our mouse model. Future studies should explore the inhibition of these factors and signaling pathways as a mechanism for restoring bone formation in the B-ALL niche. The therapeutic potential of restoring osteoblast number and function has already shown promise in murine models of acute leukemia, thus, is worthy of further investigation in B-ALL (Krevvata et al., 2014). In addition to mediating bone loss, IL-6 is considered a negative prognostic marker in many types of cancer and has been found to promote malignant cell proliferation, metastasis and anti-apoptotic pathways, therefore favoring disease progression (Kumari et al., 2016). Therefore, the impact of MSC-derived IL-6 in the B-ALL BMM should be further investigated.

The presence of pro-inflammatory factors in the B-ALL BMM is a well-documented phenomenon that appears to be reflected in the gene signature of our L-MSCs (Vilchis-Ordonez et al., 2015; Balandran et al., 2016). We observed an upregulation of genes involved in the inflammatory response which we validated by qPCR. It will be important to explore the potential implication of this L-MSC inflammatory response on leukemogenesis in future experiments. For example, pro-inflammatory cytokines can result in activation of the Jak-STAT signaling pathway (Zhao et al., 2021). Indeed, in this study, we observed an enrichment for genes in the Jak-STAT signaling pathway in L-MSCs. In AML, activation of Jak-STAT signaling in blast cells and MSCs has been reported to promote blast cell proliferation (Habbel et al., 2020). Additionally, reduced proliferation and increased apoptosis in AML-MSCs were attributed to increased Jak-STAT signaling which could be alleviated *via* Jak-STAT inhibitors (Zhang et al., 2021). Surprisingly, limited studies have explored the role of Jak-STAT activation in B-ALL-associated MSCs. Considering our findings, the effect of Jak-STAT inhibition on L-MSCs and how this may impact their B-ALL supportive function is worthy of further interrogation.

Impairment of healthy hematopoiesis is a pronounced feature of B-ALL, which is also recapitulated in our murine model (Cheung et al., 2018). As MSCs are known to be essential regulators of the normal hematopoietic stem cell (HSC) niche, we hypothesized that the hematopoietic supportive function of MSCs may be impaired by leukemia development (Pinho and Frenette, 2019). Our data revealed that LSK expansion was enhanced in the presence of L-MSCs. Interestingly, a previous study found that aged MSCs induced greater expansion of the CD34^+^ hematopoietic progenitor population *in vitro* than pediatric MSCs, which was attributed to increased IL-6 production by adult MSCs (O'Hagan-Wong et al., 2016). According to our molecular level data, *Il6* is upregulated in L-MSCs, therefore, future experiments should investigate whether this factor could be contributing to increased LSK proliferation in co-culture. This could be important, as dysregulated expansion of the hematopoietic stem and progenitor pool can lead to exhaustion of the hematopoietic system.

Degradation of the ECM is a common feature of the tumor microenvironment and has important implications for cancer invasiveness and metastasis (Winkler et al., 2020). Previously, we have shown that the BM ECM was modified by BCR-ABL1^+^ B-ALL, exemplified by a reduction in collagen type I in femurs of mice at Day 8 and Day 20 post leukemia cell injection (Cheung et al., 2018). Matrix metalloproteinases (MMPs) are ECM proteases that can degrade the BM ECM and play a vital role in the differentiation of MSCs into different lineages (Almalki and Agrawal, 2016). B-ALL cells have been shown to be capable of upregulating MMP-9 expression in BM MSCs and the expression of MMP-9 by MSCs is mediated by TNF-α-induced activation of NF-κB signaling pathways (Verma et al., 2020). While we observed an enrichment of TNF and NF-κB signaling pathways in B-ALL-associated MSCs, the expression of *Mmp9* was not significantly increased in L-MSCs in our study. However, we did observe that the expression of another member of the MMP protein family, *Mmp11*, was upregulated in L-MSCs (Log2FC = 3.15). *MMP11* has been shown to be upregulated in 15 different solid cancer types and is known to facilitate tumor invasion (Gobin et al., 2019). Additionally, our GO analyses of L-MSCs identified a downregulation in ECM-related processes, such as ‘collagen fibril organization’ and ‘ECM organization’, supporting the notion of ECM dysregulation in the BMM of B-ALL.

Finally, *DPEP1* expression has recently been identified as a negative prognostic indicator in patients with B-ALL (Zhang et al., 2020). Overexpression of this factor in B-ALL cells was shown to enhance their proliferation and survival, highlighting its potential as a therapeutic target (Zhang et al., 2020). Interestingly, *Dpep1* was one of the most significantly upregulated genes in L-MSCs in our study (Log2FC = 3.64), a finding we validated by qPCR. However, the role of BMM-derived DPEP1 in the pathogenesis of B-ALL is yet to be investigated and follow-up studies investigating the impact that L-MSC-derived DPEP1 has on leukemogenesis are therefore warranted.

It should be acknowledged that a limitation associated with the *in vitro* assessment of MSC biology is the inability of culture conditions to completely recapitulate the *in vivo* BMM. Thus, the possibility of culture induced changes to MSC biology must be considered when examining our results. Recent studies have begun to examine the effect of manipulating various cell culture conditions including oxygen concentration, 3-dimensional *versus* 2-dimensional culture and dynamic *versus* static culture on MSC biology (Tsai et al., 2011; Ejtehadifar et al., 2015; Tsai et al., 2019; Kouroupis and Correa, 2021). In the future, standardization of MSC culture methodology that best recapitulates the BMM will be essential for reducing the impact of culture on MSC biology. Ultimately, this will improve the accuracy of the *in vitro* assays used to study this cell population.

In summary, significant progress has been made over recent years in the development of therapies which target the B-ALL microenvironment (Kuek et al., 2021). However, the BMM of certain high-risk B-ALL subtypes, such as BCR-ABL1, remain under investigated. In this study, we provide important insight regarding alterations to MSC biology in the setting of high disease burden. We have demonstrated that BCR-ABL1^+^ B-ALL-associated MSCs exhibit reduced self-renewal capacity and extensive molecular alterations, indicating potential disruptions to important signaling pathways involved in inflammation, osteoblastogenesis and ECM organization *in vivo*. Together, our findings provide vital directions for future research, which include examining the cellular and molecular properties of L-MSCs across sequential timepoints of B-ALL progression to provide an understanding of how MSC biology is altered throughout leukemogenesis; performing protein level validation of the molecular findings identified; and delving into the therapeutically targetable aspects of MSC biology, which will be essential for continued improvement in clinical outcomes of patients diagnosed with B-ALL.

## Data Availability

The datasets presented in this study can be found in online repositories. The names of the repository/repositories and accession number(s) can be found below: Gene Expression Omnibus, accession number: GSE208719.
