# Supplementary material for: Characterization of mesenchymal stem cells in pre-B acute lymphoblastic leukemia
Source: Front Cell Dev Biol. 2023 Jan 20;11:1005494. doi: 10.3389/fcell.2023.1005494 (PMC9897315; doi:10.3389/fcell.2023.1005494)
Supplement: Supplementary file 2 [file DataSheet1.pdf]

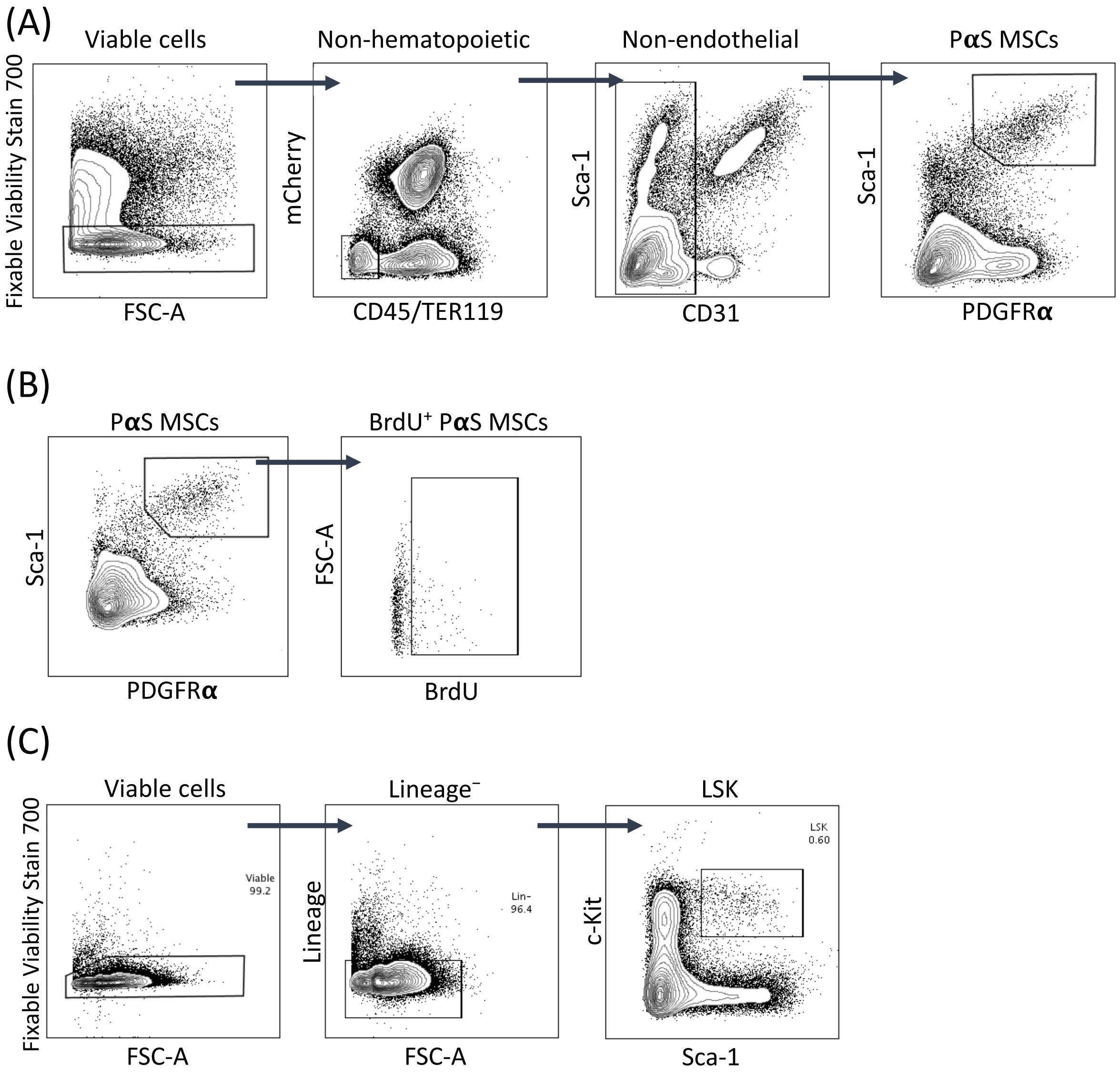

Supplementary Figure 1. Flow cytometry/fluorescence activated cell sorting (FACS) gating strategies. (A) PDGRFα^+, Sca-1$^+$ (PαS) mesenchymal stem cell (MSC) gating strategy. **(B)** Detection of BrdU$^+$ PαS MSCs by flow cytometry. **(C)** Lineage$^-$, Sca-1$^+$, c-Kit$^+$ (LSK) gating strategy.

Supplementary Methods

MSC differentiation assays

To assess the osteogenic potential of mesenchymal stem cells (MSCs), MSCs were seeded into a 96-well plate at 8500 cells/well and allowed to adhere overnight. The following day, osteogenic differentiation medium (hMSC Osteogenic Differentiation BulletKit, Lonza) was added to the wells. Cells were cultured for 13-28 days, with media changed every 3 days. After 13 days, cells were washed with phosphate-buffered saline (PBS), fixed with 4% paraformaldehyde (PFA) for 1 minute, followed by washing with a tween wash buffer (0.05% tween in PBS). Differentiating osteoblasts were stained with an alkaline phosphatase solution (SIGMA*FAST* BCIP/NBT tablet, Sigma-Aldrich, dissolved in 10ml of deionized water) for 15 minutes, followed by 2 rinses with the tween wash buffer. To confirm the capacity of mature osteoblasts to form bone nodules, cells were cultured in osteogenic media for 26-28 days. For staining of bone nodules, cells were washed with PBS and fixed with 2.5% glutaraldehyde for 20 minutes. After fixation, cells were washed with PBS once and 70% ethanol thrice. Wells were then air dried, followed by staining with Alizarin Red staining solution (1% in water, pH 4.2) for 20 minutes. After staining, cells were washed twice with 50% ethanol, followed by a third wash in 50% ethanol on a shaking platform. Finally, wells were air dried.

To assess the chondrogenic potential of MSCs, MSCs were resuspended at 45000 cells in 20µl of alpha-MEM medium and seeded into a 48-well plate to form a micromass. Cells were allowed to adhere overnight, followed by culture in chondrogenic differentiation medium (StemXvivo Chondrogenic base media and mouse supplement, R&D). Media was replaced every 3 days for 20-22 days. Following this, each chondrocyte micromass was washed with PBS and fixed with 2.5% glutaraldehyde, followed by staining with an Alcian blue solution (pH=1) to assess glycosaminoglycan content. To make the staining solution, Alcian blue powder (Sigma-Aldrich) was dissolved in 10% sulfuric acid at a concentration of 10mg/ml). After 2 hours, micromass' were washed with a de-staining solution (0.1M hydrochloric acid) followed by PBS twice.

To assess the adipogenic potential of MSCs, MSCs were seeded into a 96-well plate at 15000 cells/well and cultured in complete MesenCult media until confluence. Media was then replaced with Adipogenic Induction Medium (hMSC Adipogenic Differentiation BulletKit, Lonza). After 3 days, media was replaced with Adipogenic Maintenance Medium (BulletKit, Lonza). Three days later, cells were fixed with 4% paraformaldehyde for 1 hour, washed thrice with distilled water and stained with Oil Red O for 30 minutes. The Oil Red O staining solution was made in 99% triethyl phosphate (Sigma-Aldrich) as described previously (Kinkel et al., 2004). After 30 minutes wells were washed thrice with distilled water. Low oxygen culture conditions (5% O_2, 5% CO_2) were maintained throughout adipogenesis (Basciano et al., 2011).

For quantification, 4X images were taken from each well using a Nikon Eclipse Ti system fitted with a Nikon DS-Ri1 color camera. NISElements (V3.21, LO) imaging software was used to capture photos and the percentage of area stained by alkaline phosphatase, Oil Red O or Alcian blue was measured using ImageJ (v1.52a, National Institute of Health) according to a previously published protocol (Ng et al., 2014). For osteoblast mineralization, the number of bone nodules stained by Alizarin Red were counted per well to quantify bone formation.

References

Basciano, L., Nemos, C., Foliguet, B., de Isla, N., de Carvalho, M., Tran, N., et al. (2011). Long term culture of mesenchymal stem cells in hypoxia promotes a genetic program maintaining their undifferentiated and multipotent status. *BMC Cell Biol* 12**,** 12. doi: 10.1186/1471-2121-12-12.

Kinkel, A.D., Fernyhough, M.E., Helterline, D.L., Vierck, J.L., Oberg, K.S., Vance, T.J., et al. (2004). Oil red-O stains non-adipogenic cells: a precautionary note. *Cytotechnology* 46(1)**,** 49-56. doi: 10.1007/s10616-004-3903-4.

Ng, C.P., Sharif, A.R., Heath, D.E., Chow, J.W., Zhang, C.B., Chan-Park, M.B., et al. (2014). Enhanced ex vivo expansion of adult mesenchymal stem cells by fetal mesenchymal stem cell ECM. *Biomaterials* 35(13)**,** 4046-4057. doi: 10.1016/j.biomaterials.2014.01.081.
